# A Special Issue on the Roles of Dopamine in Neural Circuits, Genetics, and Behavior

**DOI:** 10.3390/brainsci14010020

**Published:** 2023-12-23

**Authors:** Suelen L. Boschen, Paolo S. D’Aquila, Deranda B. Lester

**Affiliations:** 1 Department of Neuroscience and Neurosurgery, Mayo Clinic, Jacksonville, FL 32224, USA; 2 Department of Biomedical Sciences, University of Sassari, 07100 Sassari, Italy; dsfpaolo@uniss.it; 3 Psychology Department, University of Memphis, Memphis, TN 38111, USA; dbrewer@memphis.edu

Over the past 80 years, research on dopamine has undergone significant evolution, reshaping our understanding of its roles in the brain and the body. Groundbreaking research from Arvid Carlsson and Oleh Hornykiewics in the brains of mammals elevated the role of dopamine from merely an intermediary in the synthesis of norepinephrine to full neurotransmitter status with critical roles in the neurobiology of movement and as a target in the development of therapies for movement disorders [1,2]. 

Subsequent research on dopaminergic receptors and pathways added depth to our understanding of dopamine’s involvement in reward, motivation, and cognitive processes, such that dopamine was identified as a key player in the pathogenesis of several mental disorders, such as schizophrenia, addiction, bipolar disorder, and, of course, Parkinson’s disease. This evolution in our understanding of dopamine not only laid the groundwork for targeted treatments for various neuropsychiatric conditions but also deepened our knowledge of how this multifaceted neurotransmitter influences human behavior and health.

The Special Issue “The role of dopamine in neural circuits” comprises a collection of original and review articles presenting the current status of research in genetic, epigenetic, molecular, and environmental factors that determine dopaminergic transmission and signaling in physiological and disease states.

Genetic variability in the dopaminergic system can contribute to the development of several neurological and psychiatric disorders, including attention-deficit and hyperactivity disorder, Autism Spectrum Disorder, Parkinson’s disease, and epilepsy [3,4,5]. Importantly, Stanfill and Cao demonstrate that demographic and phenotypic characteristics, such as age, sex, and body-mass index (BMI), are affected by dopaminergic gene expression in human brain tissue samples, offering a foundation for further exploration and potential applications in the screening, diagnosis, and treatment of neurological and psychiatric conditions. Additionally, Grzywacz et al. reveal important epigenetic modifications, specifically methylation patterns in the DAT1 gene, in cannabis-dependent individuals, suggesting that epigenetic changes may influence gene expression, which in turn affects cellular structure and function, potentially impacting overall metabolism and behavior. Such interactions are continued, as Suchaneka et al. stress the importance of considering both genetic and psychological elements when studying vulnerability to substance dependence by showing that the DRD2 gene rs1076560 variant influences certain personality traits associated with motivational behavior. These studies are in alignment with Nestler and Luscher’s hypothesis that the epigenetic remodeling of dopaminergic transmission renders cells and circuits more vulnerable to addiction [6].

In-depth study of the interplay between environmental factors, genetic changes, and the regulation of behavior through the dopaminergic system can be precisely and differentially investigated with the use of models from multiple species [7]. Moulin et al. used a Drosophila melanogaster model to demonstrate that transient dopaminergic imbalances during development can lead to overeating behaviors and potentially eating disorders later in life, particularly in the development of obesity-like hereditary traits and the factors influencing their transgenerational transmission. Additionally, Oliver et al. show that cell-specific modulation is key to the parse-out neural circuits involved in addiction and compulsive drug-taking behavior. By using a methamphetamine self-administration rat model, they demonstrated that the chemogenetic inhibition of D1-expressing medium spiny neurons in the dorsal striatum is associated with dysregulated D1R signaling and increased methamphetamine self-administration.

Finally, the roles of dopamine in motivation, learning and memory, and habit formation are discussed through the examination of dopamine’s contribution to the development and maintenance of a brain network that supports healthier lifestyles and enhances cognitive reserve. Ruiz-Tejada et al. review evidence from rodent and human studies, emphasizing the importance of dopamine and its associated mechanisms in regulating engagement in voluntary physical activity behavior. Cabib et al. propose a hypothesis that stress-related dopamine transmission is involved in the dynamic reorganization and preservation of functional networks in the adult brain, which may help individuals cope with age- and disease-related cognitive impairment. Importantly, dopaminergic transmission in the striatum and epigenetic modifications of the DRD2 gene have been recently implicated as neurobiological correlates of IQ malleability [8].

In essence, advances in the understanding of dopamine’s intricate functions in the brain and body, and its involvement in a range of neuropsychiatric and neurologic disorders, have enriched our insights into the profound impact of this neurotransmitter on behavior. This Special Issue underscores the ongoing significance of dopamine research, offering the potential to enhance our grasp of brain function, human well-being, and the continued refinement of therapeutic strategies. We extend our heartfelt gratitude to the dedicated authors who have contributed to this Special Issue, and we wish you an enlightening reading experience.
